# Complete Genome Sequence of a Novel Clinical Isolate, *Mycobacterium abscessus* Strain NOV0213

**DOI:** 10.1128/genomeA.01407-15

**Published:** 2016-01-07

**Authors:** M. A. Dymova, O. I. Alkhovik, L. S. Evdokimova, A. G. Cherednichenko, T. I. Petrenko

**Affiliations:** Federal State Institution “Novosibirsk Research Institute of Tuberculosis,” Russian Ministry of Health (NRIT), Novosibirsk, Russia

## Abstract

*Mycobacterium abscessus* is a rapid-growing species of nontuberculous mycobacteria that is frequently associated with opportunistic infections in humans. We determined the complete genome sequence of the *M. abscessus* strain NOV0213, which was isolated from a patient with tuberculosis-like disease and with various antibiotic resistances.

## GENOME ANNOUNCEMENT

*Mycobacterium abscessus* belongs to a group of rapidly growing mycobacteria (RGM). *M. abscessus* is a bacterium distantly related to the ones that cause tuberculosis and leprosy. It is part of a group of environmental mycobacteria and is found in water, soil, and dust. It has been known to contaminate medications and products, including medical devices. *M. abscessus* is the most drug-resistant mycobacterial species known and exhibits unsatisfactory treatment response rates, especially for patients with pulmonary disease (1). Different species of *M. abscessus* have different drug susceptibility and treatment outcomes. However, the genomic knowledge of *M. abscessus* is limited. In this study, we determined the whole-genome sequence, by the GS Junior system, of *M. abscessus* strain NOV0213, which was isolated from a patient with tuberculosis-like disease and with a high level of resistance to various antibiotics.

Ripoll et al. first reported the complete genome sequence of the *M. abscessus* strain, ATCC 19977 T (2). Here, we present another genome for this species, strain NOV0213, isolated from a sputum sample from a Russian patient presenting with a prolonged productive cough suggestive of a bacterial lower respiratory tract infection.

The strain NOV0213 genome was sequenced using GS Junior System. Herein, we generated 85,129 reads. The mean sequence size was 22,303.3 bp and the median sequence size was 14,671 bp. These sequences were assembled with Newbler v2.3, resulting in 204 contigs with the longest contig size of 244,067 bp. Next, Mauve was used for constructing multiple genome alignments, and then remapping reads to the assembled genome was done. The genome sequence shows a genome size 5,173,145 bp. The average G+C content of this genome sequence is 64.2%. The NCBI staff used the Prokaryotic Genome Automatic Annotation Pipeline (PGAAP) to complete the annotation; 4,845 predicted coding sequences were identified.

### Nucleotide sequence accession number.

The *M. abscessus* strain NOV0213 genome sequence and annotation data have been deposited in NCBI GenBank under the accession number CP013049.
